# Association study between genetic polymorphisms in folate metabolism and gastric cancer susceptibility in Chinese Han population: A case–control study

**DOI:** 10.1002/mgg3.633

**Published:** 2019-03-18

**Authors:** Lusha Wei, Fanglin Niu, Jiamin Wu, Fulin Chen, Hua Yang, Jing Li, Tianbo Jin, Yifei Wu

**Affiliations:** ^1^ Department of Food and Nutrition Engineering Shaanxi Normal University Xi’an China; ^2^ Key Laboratory of Resource Biology and Biotechnology in Western China (Northwest University) Ministry of Education, School of Life Sciences, Northwest University Xi’an, Shaanxi China

**Keywords:** gastric cancer, genetics polymorphisms, *MTHFR*, *MTR*, *MTRR*

## Abstract

**Background:**

Gastric cancer (GC), the second leading cause of cancer mortality behind lung cancer worldwide, is caused by both genetic and environmental factors. In this study, we evaluated the association between the genetic polymorphisms of methylenetetrahydrofolate reductase (*MTHFR*), methionine synthesis reductase (*MTR*), and methyltransferase reductase (*MTRR*) genes and ischemic stroke risk in Chinese population.

**Methods:**

A case–control study was conducted including 681 patients with GC and 756 healthy controls. Chi‐squared test/Fisher's exact test and genetic model were used to evaluate associations. Odds ratios (ORs) and 95% confidence intervals (CIs) were calculated using unconditional logistic regression.

**Results:**

In the allele model, using the chi‐square test, we found that the rs1532268 in *MTRR* with a minor allele T was significantly associated with increased risk of GC (OR = 1.24, 95% CI, 1.00–1.53; *p = *0.048). In the genetic model analysis, we identified that the single‐nucleotide polymorphism of the rs1801133 in *MTHFR* could increase the GC risk in the recessive model (OR = 1.31, 95% CI, 1.01–1.70; *p = *0.042) and log‐additive model (OR = 1.19, 95% CI, 1.02–1.38; *p = *0.025). In *MTHFR*, a strong linkage of rs2274976 and rs1801133 was detected. The haplotype “GC” in the *MTHFR* gene was found to prominently increase the risk of GC (OR = 1.26, 95% CI: 1.07–1.47; *p = *0.005). Other haplotypes did not display the correlativity.

**Conclusion:**

This study suggested that* MTR* and *MTHFR *polymorphisms may contribute to increase the risk of GC.

## 1 BACKGROUND

Gastric cancer (GC), one of the most common cancers, is the second leading cause of cancer mortality behind lung cancer worldwide. Particularly in China, an obvious clustering of geographical distribution of GC and a high mortality are estimated (Li et al., 2017; Yang, 2006). Despite remarkable decline in GC mortality is noticed, because of the poor prognosis and limited treatment options, there remains a major challenge in clinical with population growth. Smoking, high salt intake, smoking, and a familial genetic component are also recognized as predisposing factors. Additionally, hereditary genes were assessed to contribute 28% inducement by model fitting (De, Forman, & Plummer, 2003).

Genome‐wide association studies (GWASs), aimed at detecting variants at genomic loci such as single‐nucleotide polymorphisms (SNPs) that are associated with complex traits in populations, indicate new genetics insights about malignancies. Through GWAS, a number of genetic variation at different genes involved in gastric carcinogenesis and prognosis have been identified such as *XPG*, *PLCE1*, *HFE*, *ERCC5*, *EZH2*, *DOC2*, *CYP19A1*, *ALDH2*, and *CDH1* (González, Sala, & Rokkas, 2013; Xia et al., 2015). Folate, an important constituent of vegetables and fruits, has been proved to decrease the risk of colorectal, pancreatic, and esophageal cancers by cumulative evidence. Besides an inadequate folate intake, polymorphisms in folate metabolism‐related enzyme‐coding genes, which play critical roles in DNA methylation and synthesis, have been previously suggested the correlation with the risk of cancer in various sites, including gastric carcinoma. So far, in terms of GC, SNPs like rs1801133 and rs1801131 in the methylenetetrahydrofolate reductase (*MTHFR*) (OMIM: 607093) are most extensively studied in. These SNPs may decrease the activity of *MTHFR*, resulting in increased levels of homocysteine (Kumar et al., 2012). Genetic variations of 5‐methyltetrahydrofolate‐homocysteine methyltransferase reductase (*MTRR*) (OMIM: 602568) and methionine synthesis reductase (*MTR*) (OMIM: 156570) genes were recently reported mainly associated to other cancers (breast, colon, prostate, pancreatic) (Ohnami et al., 2008; Shrubsole et al., 2006; Wu, Tang, & An, 2014).

Previous studies suggested there were differences of susceptibility variants in different races. China has one of the highest GC incidences in the world (Rafiei, Mohammadian‐Hafshejani, Towhidi, Makhsosi, & Salehiniya, 2016). For evaluating the correlations between genetic polymorphisms in the folate pathway and the risk of GC in Chinese, herein, we expanded the sample scale and focused on metabolism‐related genes *MTHFR*, *MTR*, and *MTRR*. The investigation was hoped to provide theoretical foundation of the study in potential functional SNPs.

## 2 MATERIALS AND METHODS

### 2.1 Ethics statement

This investigation was conducted in accordance with the ethical standards of the Declaration of Helsinki and following national and international guidelines. Additionally, our study has been approved by the ethics committee of the first affiliated Hospital of Xi^'^an Jiaotong University. All participants were informed both in writing and verbally of the procedures and purpose of our research and signed informed consent documents were obtained from all subjects.

### 2.2 Research participates

The study groups comprised 681 patients with GC and 756 healthy controls. All the subjects were recruited from Northwestern China at the first affiliated Hospital of Xi^'^an Jiaotong University. The related clinical information, including residence, age, ethnicity, sex, dietary habits, and previous disease history were collected through a detailed questionnaire. The patients were recently diagnosed with primary GC on the basis of clinical manifestations with further confirmation by endoscopic and histopathological analysis. Cases with other types of cancers or who underwent radiotherapy or chemotherapy were excluded. Unrelated healthy individuals through strict physical examinations in the same hospital were enrolled as controls. Patients had any other form of cancer, gastritis, or gastric ulcers or had blood relatives with GC going back three generations were excluded.

### 2.3 SNP selection and genotyping

We screened the SNPs of *MTHFR*, *MTR*, and *MTRR* with over 5% minor allele frequency (MAF) and disease relevance in 1,000 genome (http://www.internationalgenome.org/). For this study, we selected seven related SNPs, according to previous studies, rs2274976 and rs1801133 in *MTHFR*, rs1805087 and rs2853522 in *MTR*,Rs1801394, rs1532268, and rs162036 in *MTRR*, for the association analysis (Jae‐Young et al., 2012).

A venous blood sample (5 ml) was collected from each participates and were stored at −80°C. Patients’ blood sample collection was implemented before radiation or chemotherapy. Genomic DNA isolation was executed using a Gold Mag‐Mini Whole Blood Genomic DNA Purification Kit (GoldMag Ltd, Xian, China). Spectrometry (DU530 UV/VIS spectrophotometer, Beckman Instruments, Fullerton, CA) was used to measure the DNA concentration. Agena MassARRAY Assay Design 4.0 software was used to design the multiplexed SNP Mass EXTEND assay and Agena MassARRAY RS1000 was used to perform SNP genotyping according to the standard protocol. Then, Agena Typer 4.0 software was applied to analyze and manage our data (Chen et al., 2017; Gabriel, Ziaugra, & Tabbaa, 2009; Tian et al., 2018).

### 2.4 Statistical analysis

We used SPSS version 19.0 software (SPSS, Chicago, IL) and Microsoft Excel (Redmond, WA) to analyze all the related data. A value of *p < *0.05 was considered as statistically significant. A chi‐squared test was used to evaluate the genotype frequencies of cases and controls. Frequencies of the variants were estimated using the Hardy–Weinberg equilibrium (HWE) (*p* value calculated by exact test) to compare the expected frequencies of the genotypes in the control groups. PLINK software (http://pngu.mgh.harvard.edu/purcell/plink/) were used to calculate the 95% confidence intervals (CIs) and odds ratios (ORs) by unconditional logistic regression analysis with adjustment for age and gender (Lin et al., 2017), in order to assess the strength for the association of genotypes and their combinations with GC risk in the four models (codominant, dominant, recessive, and log‐additive) (Jin et al., 2016; Lin et al., 2017; Liu et al., 2017). Finally, we measured the linkage disequilibrium (LD) between loci, haplotype construction, and genetic association was calculated by unconditional logistic regression. The Haploview were used to construct haplotype and genetic association at significant polymorphism loci and to estimate the pairwise LD, haplotype software (version4.2) (Barrett, Fry, Maller, & Daly, 2005).

## 3 RESULTS

### 3.1 Characteristics of the participants

A case–control study containing 681 GC patients with a mean age of 57.57 ± 10.826 and 756 healthy controls with a mean age of 52.58 ± 8.709 was performed. The general characteristics are listed in Table 1.

**Table 1:** General characteristics of the study population

Variables	Case (%)	Control (%)	Total	*p*
Total	681	756		
Gender				<0.001
Male	527 (77.4)	489 (64.7%)	1,016	
Female	154 (22.6)	267 (35.3%)	421	
Age				<0.001
Mean age ± *SD*	57.57 ± 10.826	52.58 ± 8.709		

Note

Values of *p* were calculated from a two‐sided chi‐squared test/Fisher's exact test. Value of *p ≤ *0.05 was statistically significant.

### 3.2 *The associations between SNPs and GC risk*

Basic information containing position, alleles, MAF distribution, HWE‐*p* value, ORs, 95% CIs of all the candidate SNPs is shown in Table 2. The minor allele of each SNP was assumed as a risk allele compared to the wild‐type allele and MAF was listed. The HWE‐*p* values showed none of the seven SNPs had a significant departure from the HWE. OR = 1 indicates that the factor had no effect on the disease; OR >1 means a risk factor; and OR <1 means protective factor. The *p* values were estimated by the chi‐square test. From the seven SNPs, only rs1532268 on *MTRR* with a minor allele T was significantly associated with increased risk of GC (OR = 1.24, 95% CI, 1.00–1.53; *p = *0.048).

**Table 2:** Basic information of candidate SNPs in all the individuals examined in this study

SNP ID	Gene	Position	Band	Allele (aA/B)	MAF		HWE‐*p*	OR (95% CI)	*p* value
					Case	Control			
rs2274976	*MTHFR*	11790870	1p36.22	G/A	0.059	0.065	1.000	0.90 (0.67−1.22)	0.507
rs1801133	*MTHFR*	11796321	1p36.22	T/C	0.476	0.451	0.340	1.11 (0.96−1.28)	0.173
rs1805087	*MTR*	236885200	1q43	G/A	0.08	0.1	1.000	0.78 (0.60−1.01)	0.061
rs2853522	*MTR*	236897756	1q43	A/C	0.428	0.421	0.455	1.03 (0.89−1.19)	0.698
rs1801394	*MTRR*	7870860	5p15.31	G/A	0.268	0.284	0.858	0.92 (0.78−1.09)	0.333
rs1532268	*MTRR*	7878066	5p15.31	T/C	0.153	0.128	0.071	1.24 (1.01−1.53)	0.048*
rs162036	*MTRR*	7885846	5p15.31	G/A	0.163	0.174	0.377	0.92 (0.76−1.12)	0.422

Note

Value of HWE‐*p* ≤ 0.05 was excluded.

SNP: single nucleotide polymorphism; A: minor allele; B: major allele. MAF: minor allele frequency; HWE: Hardy–Weinberg equilibrium; ORs: odds ratios; 95% CI: 95% confidence interval.

a Minor allele.

* Value of *p* ≤ 0.05 indicates statistical significance.

### 3.3 *Associations between genotype frequencies and GC risk*

The correlations between polymorphisms and GC susceptibility were analyzed based on four genetic models (codominant, dominant, recessive, and log‐additive) using logistic tests, as shown in Table 3. After adjusting for age and gender, one SNP was found to be conspicuous: rs1801133 in *MTHFR* increased the GC risk in the recessive model (OR = 1.31, 95% CI, 1.01–1.70; *p = *0.042) and log‐additive model (OR = 1.19, 95% CI, 1.02–1.38; *p = *0.025). Besides, no polymorphism displayed the association based on statistically significance.

**Table 3:** Logistic regression analysis of the association between prominent SNPs with the gastric cancer (adjusted sex and age)

Gene	SNP ID	Model	Genotype	Control, *n* (%)	Case, *n* (%)	Without adjusted		With adjusted	
						OR (95% CI)	*p*	OR (95% CI)	*p*
*MTHFR*	rs2274976	Codominant	A/A	661 (87.3)	604 (88.6)	1.00	0.740	1.00	0.990
			A/G	93 (12.3)	75 (11)	0.88 (0.64−1.22)		0.98 (0.70−1.38)	
			G/G	3 (0.4)	3 (0.4)	1.09 (0.22−5.44)		1.10 (0.21−5.70)	
		Dominant	A/A	661 (87.3)	604 (88.6)	1.00	0.470	1.00	0.940
			A/G‐G/G	96 (12.7)	78 (11.4)	0.89 (0.65−1.22)		0.99 (0.71−1.38)	
		Recessive	A/A‐A/G	754 (99.6)	679 (99.6)	1.00	0.900	1.00	0.910
			G/G	3 (0.4)	3 (0.4)	1.11 (0.22−5.52)		1.10 (0.21−5.70)	
		Log‐addition	—	—	—	0.90 (0.67−1.22)	0.510	0.99 (0.72−1.36)	0.960
*MTHFR*	rs1801133	Codominant	C/C	234 (30.9)	192 (28.3)	1.00	0.430	1.00	0.076
			C/T	361 (47.8)	327 (48.2)	1.10 (0.87−1.41)		1.14 (0.88−1.47)	
			T/T	161 (21.3)	160 (23.6)	1.21 (0.91−1.62)		1.42 (1.05−1.93)	
		Dominant	C/C	234 (30.9)	192 (28.3)	1.00	0.270	1.00	0.096
			C/T‐T/T	522 (69)	487 (71.7)	1.14 (0.91−1.43)		1.22 (0.96−1.55)	
		Recessive	C/C‐C/T	595 (78.7)	519 (76.4)	1.00	0.300	1.00	0.042*
			T/T	161 (21.3)	160 (23.6)	1.14 (0.89−1.46)		1.31 (1.01−1.70)	
		Log‐addition	—	—	—	1.10 (0.95−1.27)	0.190	1.19 (1.02−1.38)	0.025*
*MTR*	rs1805087	Codominant	A/A	611 (80.9)	577 (84.6)	1.00	0.170	1.00	0.290
			A/G	137 (18.1)	101 (14.8)	0.78 (0.59−1.03)		0.86 (0.64−1.15)	
			G/G	7 (0.9)	4 (0.6)	0.61 (0.18−2.08)		0.46 (0.13−1.65)	
		Dominant	A/A	611 (80.9)	577 (84.6)	1.00	0.065	1.00	0.210
			A/G‐G/G	144 (19.1)	105 (15.4)	0.77 (0.59−1.02)		0.83 (0.62−1.11)	
		Recessive	A/A‐A/G	748 (99.1)	678 (99.4)	1.00	0.460	1.00	0.240
			G/G	7 (0.9)	4 (0.6)	0.63 (0.18−2.16)		0.47 (0.13−1.69)	
		Log‐addition	—	—	—	0.78 (0.60−1.01)	0.059	0.82 (0.63−1.08)	0.160
*MTR*	rs2853522	Codominant	C/C	247 (32.9)	224 (32.9)	1.00	0.780	1.00	0.950
			A/C	377 (50.1)	332 (48.8)	0.97 (0.77−1.23)		1.01 (0.79−1.29)	
			A/A	128 (17)	125 (18.4)	1.08 (0.79−1.46)		1.05 (0.76−1.45)	
		Dominant	C/C	247 (32.9)	224 (32.9)	1.00	0.980	1.00	0.840
			A/C‐A/A	505 (67.2)	457 (67.1)	1.00 (0.80−1.24)		1.02 (0.81−1.29)	
		Recessive	C/C‐A/A	624 (83)	556 (81.6)	1.00	0.510	1.00	0.760
			A/C	377 (50.1)	332 (48.8)	1.10 (0.84−1.44)			
		Log‐addition	—	—	—	1.03 (0.88−1.19)	0.730	1.02 (0.88−1.20)	0.760
*MTRR*	rs1801394	Codominant	A/A	388 (51.4)	366 (53.7)	1.00	0.600	1.00	0.600
			A/G	305 (40.4)	266 (39.1)	0.92 (0.74−1.15)		0.93 (0.74−1.17)	
			G/G	62 (8.2)	49 (7.2)	0.84 (0.56−1.25)		0.82 (0.54−1.25)	
		Dominant	A/A	388 (51.4)	366 (53.7)	1.00	0.370	1.00	0.410
			A/G‐G/G	367 (48.6)	315 (46.3)	0.91 (0.74−1.12)		0.91 (0.73−1.14)	
		Recessive	A/A‐A/G	693 (91.8)	632 (92.8)	1.00	0.470	1.00	0.410
			G/G	62 (8.2)	49 (7.2)	0.87 (0.59−1.28)		0.84 (0.56−1.27)	
		Log‐addition	—	—	—	0.92 (0.78−1.08)	0.310	0.92 (0.77−1.09)	0.320
*MTRR*	rs1532268	Codominant	C/C	581 (76.8)	498 (73)	1.00	0.150	1.00	0.280
			C/T	157 (20.8)	159 (23.3)	1.18 (0.92−1.52)		1.14 (0.88−1.49)	
			T/T	18 (2.4)	25 (3.7)	1.62 (0.87−3.00)		1.55 (0.81−2.94)	
		Dominant	C/C	581 (76.8)	498 (73)	1.00	0.094	1.00	0.180
			C/T‐T/T	175 (23.1)	184 (27)	1.23 (0.97−1.56)		1.19 (0.92−1.52)	
		Recessive	C/C‐C/T	738 (97.6)	657 (96.3)	1.00	0.150	1.00	0.210
			T/T	18 (2.4)	25 (3.7)	1.56 (0.84−2.89)		1.50 (0.79−2.84)	
		Log‐addition	—	—	—	1.22 (0.99−1.49)	0.058	1.18 (0.96−1.46)	0.120
*MTRR*	rs162036	Codominant	A/A	519 (68.7)	477 (70)	1.00	0.550	1.00	0.620
			A/G	210 (27.8)	187 (27.5)	0.97 (0.77−1.22)		0.97 (0.76−1.24)	
			G/G	26 (3.4)	17 (2.5)	0.71 (0.38−1.33)		0.72 (0.37−1.40)	
		Dominant	A/A	519 (68.7)	477 (70)	1.00	0.590	1.00	0.640
			A/G‐G/G	236 (31.3)	204 (30)	0.94 (0.75−1.18)		0.95 (0.75−1.20)	
		Recessive	A/A‐A/G	729 (96.6)	664 (97.5)	1.00	0.290	1.00	0.340
			G/G	26 (3.4)	17 (2.5)	0.72 (0.39−1.33)		0.73 (0.38−1.40)	
		Log‐addition	—	—	—	0.92 (0.76−1.12)	0.420	0.93 (0.76−1.14)	0.480

Note

Values of *p* were calculated by unconditional logistic regression adjusted for age and gender.

SNP: single nucleotide polymorphism; ORs: odds ratios; 95% CI: 95% confidence interval.

* Value of *p* ≤ 0.05 indicates statistical significance.

### 3.4 *Associations between haplotype analyses and GC risk*

**Figure 1:**
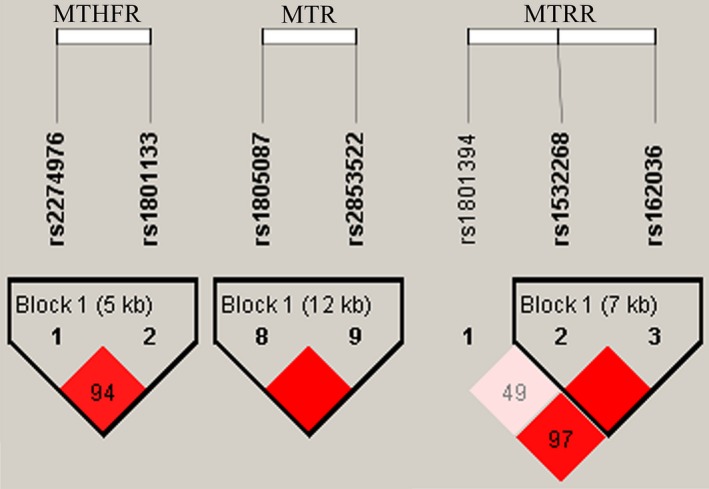
Linkage disequilibrium (LD) plots

Haplotype associations were detected based on pairwise LD of *MTHFR*, *MTR*, and *MTRR*. D’ value was calculated for the LD of pairwise SNPs, and the adjacent SNPs with pairwise D’ = 0.98 were classified in the same block. LD is indicated by bright red (very strong: LOD ≥2, D’ = 1) and light red (LOD ≥2, D’ < 1). As shown in Figure 1, rs2274976 with rs1801133, and rs1805087 with rs2853522 consisted of one block in *MTHFR* and *MTR*, respectively. In *MTR*, a strong linkage of rs1532268 and rs162036 was detected. As shown in Table 4, we used the chi‐square test and logistic test to analyze the haplotype. Haplotype “GC” in the *MTHFR* gene was found to prominently increase the risk of GC (OR = 1.26, 95% CI, 1.07–1.47; *p = *0.005). Other haplotypes did not display the correlativity.

**Table 4:** Haplotype frequencies and the association with gastric cancer

SNPs	Haplotype	Freq (case)	Freq (control)	Without adjusted		With adjusted	
				OR (95% CI)	*p* a	OR (95% CI)	*P* a
rs2274976/rs1801133	AC	0.517	0.545	1.00	—	1.00	—
	GC	0.424	0.390	1.16 (1.00–1.35)	0.057	1.26 (1.07–1.47)	0.005*
	GT	0.052	0.062	0.84 (0.60–1.18)	0.320	0.94 (0.66–1.33)	0.710
rs1805087/rs2853522	AC	0.493	0.481	1.00	—	1.00	—
	AA	0.427	0.420	0.99 (0.85–1.16)	0.940	1.00 (0.85–1.17)	0.990
	GC	0.080	0.100	0.78 (0.60–1.02)	0.069	0.82 (0.62–1.09)	0.170
rs1532268/rs162036	CA	0.684	0.699	1.00	—	1.00	—
	CG	0.162	0.174	0.96 (0.78–1.17)	0.660	0.96 (0.78–1.18)	0.680
	TA	0.153	0.128	1.20 (0.98–1.48)	0.078	1.17 (0.94–1.45)	0.150

Note

SNP: single nucleotide polymorphism; ORs: odds ratios; 95% CI: 95% confidence interval.

a Adjusted by sex and age.

* Value of *p* ≤ 0.05 indicates statistical significance.

### 3.5 Stratification analysis

To further explore the gender influence on the potential GC susceptibility of selected polymorphisms in *MTHFR*, *MTR*, and *MTRR* genes, we performed the same statistical analysis in males and females, separately. In the allele model, stratified by gender, we discovered two SNPs (rs1805087 and rs1532268) were notably correlated with a decreased risk of GC in male, respectively. The rs1805087 in *MTR*, a protective factor, was associated with a decreased risk of GC (OR = 0.66, 95% CI, 0.48–0.91; *p = *0.01), and on the contrary, the rs1532268 in *MTRR* showed an increased risk to the GC susceptibility (OR = 1.31, 95% CI, 1.02–1.68; *p = *0.036) **(**Table 5 **)**.

**Table 5:** Basic analysis of association between sex and the risk of gastric cancer

SNP	Allele (A^a^/B)	Male				Female			
		MAF (case)	MAF (control)	OR (95% CI)	*p* value	MAF (case)	MAF (control)	OR (95% CI)	*p* value
rs2274976	G/A	0.053	0.056	0.94 (0.64−1.38)	0.758	0.081	0.082	0.98 (0.59−1.64)	0.950
rs1801133	T/C	0.462	0.440	1.09 (0.92−1.30)	0.314	0.526	0.472	1.24 (0.94−1.64)	0.131
rs1805087	G/A	0.070	0.102	0.66 (0.48−0.91)	0.010*	0.114	0.096	1.21 (0.77−1.91)	0.413
rs2853522	A/C	0.422	0.419	1.01 (0.85−1.21)	0.885	0.448	0.424	1.10 (0.83−1.46)	0.503
rs1801394	G/A	0.279	0.301	0.90 (0.74−1.09)	0.281	0.227	0.252	0.87 (0.63−1.22)	0.423
rs1532268	T/C	0.159	0.127	1.31 (1.02−1.68)	0.036*	0.133	0.130	1.03 (0.68−1.56)	0.887
rs162036	G/A	0.163	0.167	0.97 (0.77−1.22)	0.787	0.162	0.186	0.85 (0.58−1.23)	0.385

Note

Value of HWE‐*p* ≤ 0.05 was excluded. Value of *p* was adjusted by age.

SNP: single nucleotide polymorphism; MAF: minor allele frequency; ORs: odds ratios; 95% CI: 95% confidence interval.

a Minor allele.

* Value of *p* ≤ 0.05 indicates statistical significance.

In the genetic model (Table 6), no significant association was found in females. However, in male, the rs2274976 in MTHFR was correlated with 1.50‐fold increased risk of GC (OR = 1.50, 95% CI, 1.07–2.10; *p = *0.018) in the recessive model, while the rs1805087 in MTR was correlated with 0.65‐fold decreased risk (OR = 0.65, 95% CI, 0.45–0.94; *p = *0.021) in the dominant model. Besides, as for haplotype analysis, no remarkable SNPs were related neither in males nor in females.

**Table 6:** Analysis of SNPs and gastric cancer risk in males and females based on logistic tests

SNP ID	Model	Genotype	Male				Female			
			Control, *n *(%)	Case, *n* (%)	OR (95% CI)	*p*	Control, *n* (%)	Case, *n* (%)	OR (95% CI)	*p*
rs1805087 (*MTR*)	Codominant	A/A	394 (80.7)	457 (86.7)	1.00	0.063	216 (81.2)	119 (77.3)	1.00	0.520
		A/G	88 (18.0)	66 (12.5)	0.66 (0.45−0.96)		49 (18.4)	35 (22.7)	1.24 (0.76−2.04)	
		G/G	6 (1.2)	4 (0.8)	0.49 (0.12−1.96)		1 (0.4)	0 (0)	0.00 (0.00‐NA)	
	Dominant	A/A	394 (80.7)	457 (86.7)	1.00	0.021*	216 (81.2)	119 (77.3)	1.00	0.420
		A/G‐G/G	94 (19.3)	70 (13.3)	0.65 (0.45−0.94)		50 (18.8)	35 (22.7)	1.22 (0.75−2.00)	
	Recessive	A/A‐A/G	482 (98.8)	523 (99.2)	1.00	0.350	265 (99.6)	154 (100)	1.00	0.450
		G/G	6 (1.2)	4 (0.8)	0.52 (0.13−2.08)		1 (0.4)	0 (0)	0.00 (0.00‐NA)	
	Log‐addition				0.67 (0.48−0.94)	0.019*			1.20 (0.74−1.94)	0.470

Note

Value of *p* was adjusted by age.

SNP: single nucleotide polymorphism; ORs: odds ratios; 95% CI: 95% confidence interval.

* Value of *p* ≤ 0.05 indicates statistical significance.

## 4 DISCUSSION

It is well documented that individual susceptibility to the development of cancer can vary. Despite the fact that the polymorphism in folate pathway was involved in carcinogenesis, the conclusions of case–control analysis were inconsistent among different ethnic groups. We researched the associations between SNPs on folate metabolism‐related genes—*MTHFR*, *MTR*, and *MTRR*—and the risk of GC. In view of the whole collected subjects, rs1532268 and rs1801133 were detected as risk factors. Haplotype GC constructed by polymorphisms rs2274976 and rs1801133 in MTHFR gene was associated with increased GC risk. In addition, by further stratification analysis, gender distribution had differences between the cases and controls. In male population, rs1532268 increased the GC risk and rs1805087 was recognized as a protective factor.

The correlation of a common polymorphism of *MTHFR*, rs1801133 (C677T), with GC risk has been analyzed repeatedly. In addition, its GC susceptibility was determined positively only in Asian individuals. A subgroup meta‐analysis by Lina Sun *et al *showed an increased risk in the Asians, but not in North American or European populations (Sun, Liang, Yuan, Jiangqi, & Jiang, 2014). Long Chen et al. identified a statistically significantly elevated risk of GC in Asian *MTHFR* C677T polymorphism populations. E Zintzaras carried out a 1,584/2,785 cases/controls study and recognized that evidence of the association was mainly in East Asian, and in Caucasians, the value was not significant. Additionally, the "T/T" genotype of rs1801133 was associated with a higher GC risk than the "C/C" genotype, which is similar to that reported in our case‐control studies (Zintzaras, 2006).

*MTR* encodes enzyme catalyzing the methylation of homocysteine to methionine with simultaneous conversion of 5‐methyl‐THF to tetrahydrofolate (THF), which is essential for DNA synthesis. The present study indicated rs1805087 as a predictor for GC. Evidences have been provided that G allele of *MTR* A2756G polymorphism was associated with multiple diseases, such as autism, head, and neck cancer (Galbiatti et al., 2010; Haghiri, Mashayekhi, Bidabadi, & Salehi, 2016). The GG genotype was reported to affect the DNA hypomethylation and individuals who carried 2756GG showed a lower frequency of CpG island hypermethylation in tumor suppressor genes (Goode, Potter, Bigler, & Ulrich, 2004; Zhao et al., 2013).

*MTRR* is a key enzyme in the homocysteine/methionine metabolic pathway. For functional polymorphism in *MTRR*, rs1801394 was commonly estimated. An interaction between *MTRR* A66G polymorphism (rs1801394) and colorectal cancer susceptibility was identified (Wang, Li, Wang, He, & Xi, 2017). An association between the A66G gene polymorphism and LC risk in a Turkish population was also been suggested (Aksoy‐Sagirli, Erdenay, Kaytan‐Saglam, & Kizir, 2017). Jae‐Young Yoo *et al* also reported that rs1801394 was associated with GC risk in Koreans by genotype analysis (Yoo et al., 2012; Yuan et al., 2012). Interestingly, in the current population, no positive association was observed, and the linkage of rs1532268 in *MTRR* and GC risk was first revealed in the Han Chinese population.

Nevertheless, there are limitations that need to be noticed in the current study. First of all, the inherent selection bias and information bias were inevitable problems, because the subjects of investigation were enrolled from the identical hospital. Second, the number of cases in our study was not large, which cannot preclude false‐negative results and cannot be extrapolated to other populations. So, larger sample size and further confirmation in other ethnic populations are needed for further verification. Despite the limitations mentioned above, the results of our present study provided scientific evidence of genes—*MTHFR*, *MTR* and *MTRR*—with the risk of GC in the future studies.

## 5 CONCLUSION

To sum up, the results of our study indicate that the rs1532268 and rs1801133 polymorphisms are potential risk factors for the development of GC in the Chinese population. rs1805087 decreased GC risk in Chinese males. For our assessment, no interaction appeared in female population. Published data demonstrated that the worldwide incidence of GC is higher in men than in women. The reason is unclear. Presumably, endogenous ovarian sex hormones may lower GC incidence in women (Duell et al., 2010; Yu, He, & Guo, 2014). Our current research is fundamental, and more functional studies are required to dig deeper.

## CONFLICTS OF INTEREST

The authors have no conflicts of interest to disclose.
